# Single-cell genome analysis of a feminizing Wolbachia in Eurema butterflies reveals a shared origin with male-killing Wolbachia

**DOI:** 10.1099/mgen.0.001578

**Published:** 2025-11-28

**Authors:** Hiroshi Arai, Yohei Nishikawa, Tatsuro Konagaya, Masato Kogawa, Masako Kifushi, Haruko Takeyama, Hisashi Anbutsu, Daisuke Kageyama

**Affiliations:** 1 Institute of Agrobiological Sciences, National Agriculture and Food Research Organization (NARO), 1-2 Owashi, Tsukuba, Ibaraki, 305-8634, Japan; 2 Institute of Infection, Veterinary and Ecological Sciences, University of Liverpool, Crown Street, Liverpool, L69 7ZB, UK; 3 Biomanufacturing and Process Research Center (BPRC), National Institute of Advanced Industrial Science and Technology (AIST), 1-1-1 Higashi, Tsukuba, Ibaraki, 305-8566, Japan; 4 Computational Bio Big-Data Open Innovation Laboratory (CBBD-OIL), AIST, 3-4-1 Okubo, Shinjuku-ku, Tokyo, 169-8555, Japan; 5 Research Organization for Nano and Life Innovation, Waseda University, 513 Wasedatsurumaki-cho, Shinjuku-ku, Tokyo, 162-0041, Japan; 6 Nara University of Education, Takabatake-cho, Nara, 630-8528, Japan; 7 Department of Life Science and Medical Bioscience, Waseda University, 2-2 Wakamatsu-cho, Tokyo, 162-8480, Japan; 8 Institute for Advanced Research of Biosystem Dynamics, Waseda Research Institute for Science and Engineering, Graduate School of Advanced Science and Engineering, Waseda University, 3-4-1 Okubo, Shinjuku-ku, Tokyo, 169-8555, Japan; 9 Bioproduction Research Institute, AIST, 1-1-1 Higashi, Tsukuba, Ibaraki, 305-8566, Japan; 10 Cellular and Molecular Biotechnology Research Institute, AIST, 2-3-26 Aomi, Koto-ku, Tokyo, 135-0064, Japan

**Keywords:** feminization, *oscar*, phage, single-cell, *Wolbachia*

## Abstract

*Wolbachia* is a ubiquitous endosymbiont in arthropods that is maternally transmitted and affects host reproduction in various ways. Among these, skewing the host sex ratio towards females, either by killing males (male killing) or producing exclusively functional females (feminization or parthenogenesis), is considered advantageous for *Wolbachia*. In the butterfly *Eurema mandarina*, individuals harbouring the *Wolbachia* strain *w*Fem exclusively produce female offspring. This occurs through a two-step mechanism in which *Wolbachia* blocks the transmission of the Z chromosome from Z0 females and feminizes the resultant Z0 offspring. Given the unique characteristics of *w*Fem, understanding its genomic features is crucial to uncover the evolution and mechanisms of *Wolbachia*-induced reproductive manipulation. However, technical challenges in isolating *w*Fem from co-infecting, closely related, non-male-killing/non-feminizing *w*CI *Wolbachia* strain have hindered genomic analyses of *w*Fem. In this study, we established a closed circular genome of *w*Fem by developing a series of *Wolbachia* purification, cell sorting and single-cell genome sequencing techniques. *w*Fem genome, ~1.3 Mb in size, specifically encodes male-killing gene homologues (*Em-oscar* and *wmk*) and other putative virulence factors that are absent in *w*CI. In addition, *w*Fem carried prophage elements that showed high similarity to previously characterized male-killing-associated prophages in *Wolbachia* strains. This study highlights the shared functional genomic features between feminizing and male-killing *Wolbachia* in Lepidoptera and suggests a mechanistic link between these two *Wolbachia*-induced reproductive phenotypes.

**Impact Statement**

Among the maternally inherited intracellular bacteria that are widespread in arthropods, some strains manipulate host reproduction by skewing the sex ratio towards females. In a butterfly species, a unique *Wolbachia* strain (*w*Fem) alters sex chromosome inheritance and sex determination, leading to all-female offspring through feminization. However, isolating *w*Fem has been technically challenging, hindering genomic analysis. In this study, we applied single-cell genomics to overcome these obstacles and successfully resolved the genomic architecture of *w*Fem, demonstrating the utility of this approach for studying unculturable endosymbionts. Notably, we found that *w*Fem encodes key effector genes known to be associated with male killing. Our findings suggest a mechanistic link between male killing and feminization and support a model in which horizontal gene transfer via bacteriophages has contributed to the evolution of novel selfish reproductive traits in symbionts.

## Data Summary

The authors confirm all supporting data, code and protocols have been provided within the article or through supplementary data files. High-throughput sequencing data are also available under the accession numbers DRR599209 (DRA), PRJDB18878 (BioProject) and SAMD00823266 (BioSample). The *w*Fem genome data are accessible under the accession number AP038754.

## Introduction

Heritable endosymbiotic microbes are common in insects and other arthropods and have various effects on host biology [1,3]. Some endosymbionts manipulate the host’s reproductive system and distort sex ratios to enhance transmission. Maternally transmitted endosymbiotic bacteria belonging to the genus *Wolbachia* (*Alphaproteobacteria*) are estimated to be present in at least 40% of all insect species, constituting one of the most widespread endosymbiont genera on the planet [2,4]. *Wolbachia* manipulates host reproduction in various ways, including cytoplasmic incompatibility, parthenogenesis, male killing and feminization [2]. Among these strategies, feminization makes all offspring functional females, thereby enhancing the spread of maternally transmitted *Wolbachia* within the population and skewing the population sex ratio towards females [5].

In contrast to the widespread occurrence of male killing, feminization is much less common and is observed only in a few insect and isopod species [6,9]. In the woodlouse *Armadillidium vulgare* (Isopoda), maleness is determined by a sex hormone secreted by the androgenic gland in genetic males; feminizing *Wolbachia* disrupts the development of this androgenic gland to convert genetic males into functional females [8,9]. This disruption results in the failure of male differentiation and development of functional ovaries, possibly achieved by interfering with hormone signalling in the host [8]. In contrast to isopods, sex determination in insects does not involve sex hormones and is determined through a sex-determining gene cascade consisting of transcriptional regulators with sex-specific transcription or isoforms [10]. In the butterfly *Eurema mandarina* (Lepidoptera), the *Wolbachia* strain *w*Fem induces female-type sex determination in the host [11,12], and removal of *w*Fem results in high mortality and/or intersex phenotypes [6,11, 13]. Notably, the fact that male moths (such as *Ostrinia scapulalis* and *Homona magnanima*) infected with male-killing *Wolbachia* show ‘feminized’ (i.e. female-type) sex determination suggests a mechanistic link between the feminization and male-killing phenotypes in Lepidoptera [14,16]. However, in the leafhopper *Zyginidia pullula*, the effects of *Wolbachia* on sex-specific gene cascades are not apparent [17], and *Wolbachia*-infected X0 females produce X0 and XX individuals after mating with XX males, both of which are functional females [7]. The mechanism underlying feminization in this system is associated with DNA methylation [18]. Although recent studies have identified the causes or candidate factors of *Wolbachia*-induced cytoplasmic incompatibility (*cifA* and *cifB*) [19,20], parthenogenesis (*piff* and *pif*) [21,22] and male killing (*wmk* and *oscar*) [23,24], the factors involved in feminization remain unclear (acronyms of *Wolbachia* strains and genes are summarized in Table S1, available in the online Supplementary Material).

Our focal system is the *Eurema* butterfly carrying the feminizing *Wolbachia w*Fem. In most populations across the Japanese Archipelago, except in the northernmost regions, 100% of *E. mandarina* butterflies are infected with the cytoplasmic incompatibility-inducing *Wolbachia* strain *w*CI [25]. On the southern islands of Japan, such as Tanegashima and Okinawa, some *E. mandarina* females harbour the *w*Fem strain alongside *w*CI and exhibit a Z0 sex chromosome constitution, in contrast to those with only *w*CI, which display a ZW constitution. The presence of *w*Fem disrupts the maternal transmission of the Z chromosome, leading to the generation of Z0 embryos (after fertilization between Z-lacking ova and Z-bearing sperm) [11]. These Z0 individuals are then feminized by *w*Fem and become reproductively functional females. Given the unique characteristics of the *w*Fem strain, genome sequencing is crucial for understanding the mechanisms and evolutionary processes underlying *Wolbachia*-induced novel phenotypes. However, *Eurema* butterflies that are singly infected with *w*Fem are rarely found in nature. Attempts to isolate *w*Fem from co-infecting *w*CI by making a dilution series and trans-infecting such diluted solutions into cultured cells have not been successful (D. Kageyama, personal observation). Moreover, the high genetic similarity between *w*CI and *w*Fem, both members of supergroup B, hinders the construction of the *w*Fem genome without prior isolation [16].

Recently, we successfully generated the genomes of three *Wolbachia* strains (*w*Hm-a, *w*Hm-b and *w*Hm-c) co-infecting the *Homona* moth, using single-cell genomics [26]. Building on this technique, we aimed to establish the complete genome of *w*Fem and discuss the evolution of feminizing *Wolbachia* and the potential mechanisms underlying *Wolbachia*-induced phenotypes.

## Methods

### Insects

An *E. mandarina* female co-infected with *w*Fem and *w*CI was collected from Tanegashima Island, Kagoshima, Japan, in 2023 to establish an iso-female line. In the laboratory, females were allowed to lay eggs on fresh leaves of *Lespedeza cuneata* (Fabales: Fabaceae) in a net cage with a 10% sucrose solution. The artificial diet for larvae was prepared by mixing leaf powder of *Albizia julibrissin* (Fabales: Fabaceae) with Insecta F-II (Nihon‐Nosan, Yokohama, Japan). Insects were reared under a 16 h/8 h light/dark photoperiod at 25 °C. After eclosion, *w*Fem-infected females were mated with males from a normal sex ratio line free of *w*Fem.

### Single-cell genome sequencing

*Wolbachia* cells were purified from six female pupae of the iso-female line following the procedure described by Arai *et al.* [26], with several modifications. Briefly, each pupa was homogenized in 1,000 µl of IPL-41 Insect Medium (1×) (Gibco, MA, USA) using a sterilized pestle. The homogenates were centrifuged at 4 °C and 3,000 ***g*** for 5 min, and the supernatants were passed through 5.0 and 1.2 µm filters (Sartorius AG, Göttingen, Germany). The filtrates were centrifuged at 4 °C and 18,000 ***g*** for 20 min, and the pellets were resuspended into 1,000 µl of IPL-41 Insect Medium (1×) (Gibco) and centrifuged at 4 °C and 18,000 ***g*** for 20 min. The resulting pellets were resuspended in 500 µl of PBS buffer (Gibco), passed through a 5.0 µm filter (Sartorius AG) to remove aggregated cells and subjected to downstream assays.

A cell suspension (7.0×10^3^ cells µl^−1^) with 1.5% ultra-low gelling temperature agarose A5030 (Sigma-Aldrich) was prepared, and 30-µm diameter droplets were generated to achieve 0.1 cell per droplet. Cell lysis and whole-genome amplification were performed as described by Chijiiwa *et al.* [27] and Nishikawa *et al.* [28]. DNA amplified by whole-genome amplification was detected on gel beads using 1×SYBR Green stain (Thermo Fisher Scientific). Fluorescence-positive (i.e. DNA-positive) gel beads (*n*=768) were isolated on 2×384 plates using a BD FACSMelody Cell Sorter (BD Biosciences). Each gel bead was directly subjected to library preparation using an xGen DNA Library Prep Kit (IDT) and sequenced using NextSeq with NextSeq 1000/2000 P2 Reagents (Illumina, CA, USA) under 150 bp paired-end conditions. Genome data from each cell were assembled using SPAdes [29], and the completeness of the assemblies was verified using CheckM [30]. Furthermore, *w*Fem-derived assemblies (single-amplified genome) were identified based on the homologies between the assemblies and marker genes of *w*CI and *w*Fem [*wsp* (AB592926.1, AB592927.1), *hcpA* (AB592911.1, AB592913.1), *gatB* (AB592901.1, AB592903.1), *coxA* (AB592906.1, AB592908.1), *ftsZ* (AB592916.1, AB592918.1), *fbpA* (AB592921.1, AB592923.1) and the *w*Fem-specific *Em-oscar* gene] using blastn.

### Genome construction and annotations

NextSeq raw read data derived from 40 *w*Fem libraries showing over 90% completeness in their initial assemblies were concatenated for downstream assays. To identify the *w*Fem-derived long reads, Illumina data were mapped against the Nanopore data from *w*CI- and *w*Fem-infected BmN-4 cells [16] using Minimap2 [31]. Nanopore reads with more than 30% coverage were extracted using SAMtools [32] and SeqKit [33]. The extracted *w*Fem-derived Nanopore and NextSeq reads were assembled using Unicycler v0.5.0 [34]. The *w*Fem genome was annotated using DFAST [35]. Putative virulence factors and proteins with a signal peptide were predicted using EffectiveDB [36] and SignalP 5.0 (https://services.healthtech.dtu.dk/services/SignalP-5.0/), respectively. Additionally, the functional analysis of the proteins (i.e. domain predictions and gene ontology annotations) was conducted using InterPro (https://www.ebi.ac.uk/interpro/) and HMMER Scan (https://www.ebi.ac.uk/Tools/hmmer/search/hmmscan). Prophage insertions were annotated using PHASTEST [37]. Homologies among the *w*Fem-encoded genes and the phenotype-associated *Wolbachia* genes *cifA* and *cifB* [19,20, 38], *wmk* [23,26], *oscar* [16,24, 26], *pif* [21] and *piff* [22] were analysed using blast. Domain structures of these *w*Fem-encoded potential reproductive-manipulating factors were analysed using InterPro (https://www.ebi.ac.uk/interpro/), and their structural integrity was assessed for evidence of truncation, fragmentation or other disruptive mutations.

### Comparative genomics and phylogenetic analysis

The genome structures of *w*CI (AP028951, reported by Arai *et al.* [16]) and *w*Fem were compared using the d-Genies web server (https://dgenies.toulouse.inra.fr/). Homologies between the MK-associated prophage WO*w*Hm-t76 region of *w*Hm-t [26] and the *w*Fem genome were visualized using TBtools [39]. The average nucleotide identity (ANI) among *Wolbachia* genomes was calculated using pyani (https://github.com/widdowquinn/pyani).

Single-copy genes conserved among *Wolbachia* strains (Table S1) were identified using OrthoFinder [40] and concatenated, aligned and trimmed using SeqKit [33], MAFFT [41] and trimAl [42], respectively. Phylogenetic trees of the concatenated sequences of *Wolbachia* strains were constructed using maximum likelihood with bootstrap resampling of 1,000 replicates using IQ-TREE [43] and visualized using the iTOL web server (https://itol.embl.de/). The presence or absence of *oscar* homologues in male killing and feminizing *Wolbachia* strains used for the phylogenetic analysis was determined based on previous studies [16,24, 26, 44, 45] and manual blastp searches against all known *oscar* sequences, together with InterPro-based domain searches for ankyrin repeats and proteinase domains – characteristic features of Oscar proteins. Phylogenetic trees of the CifB and Oscar proteins were visualized using the same procedures.

## Results and discussion

### Single-cell genomics and phylogeny of *w*Fem

Of the 768 cell genomes sequenced and assembled, 551 were confirmed to belong to *w*Fem, based on their marker gene sets (CheckM-based max-completeness: 99.8%; >90% completeness: 63 cells and >80% completeness: 112 cells). In addition, 100 cells were derived from *w*CI (max-completeness: 95.2%; >90% completeness: 4 cells and >80% completeness: 17 cells), and the remaining 117 cells consisted of contaminants and unassigned *Wolbachia* strains that did not show any marker genes for *w*Fem or *w*CI (Table S2). These results suggest that our droplet-based single-cell genomics platform allows the collection of high-quality single-cell amplified genomes of co-infected, unculturable and low-density endosymbionts.

**Fig. 1.**
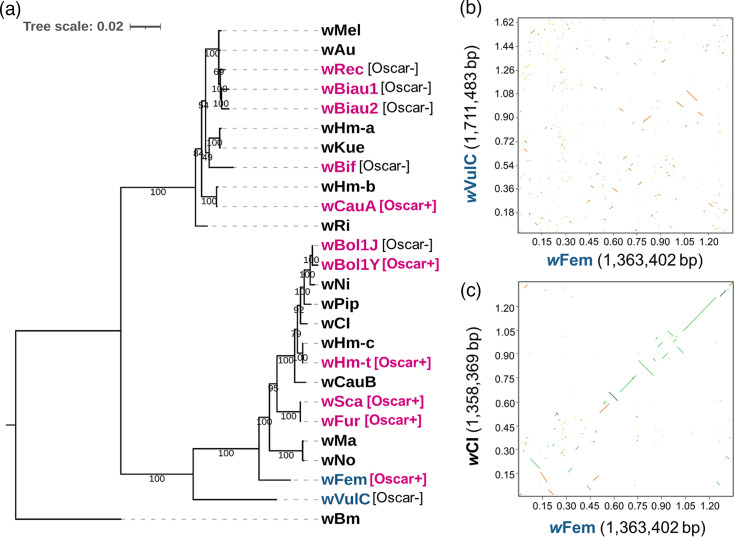
Comparative genomics of *w*Fem with other *Wolbachia* strains. (**a**) Phylogenetic tree based on the single-copy genes shared by *Wolbachia* strains. Male-killing and feminizing *Wolbachia* strains are marked in magenta and blue, respectively. Oscar+, positive for Oscar homologues; Oscar−, negative for Oscar homologues. Dot plots comparing (**b**) *w*VulC vs. *w*Fem genomes and (**c**) *w*CI vs. *w*Fem genomes. Homologous genomic regions are highlighted in green (>80% identity), yellow (50–80% identity) or orange (30–50% identity) based on their identity percentages.

*w*Fem-derived high-quality Illumina data (3.81 Gb, 25,231,920 reads and 150 bp average length, derived from 40 cells showing >90% completeness) and Nanopore data (17.2 Mb, 5,673 reads and 3,018 bp average length) were used to reconstruct a closed genome of *w*Fem consisting of a circular chromosome (1,363,402 bp in length) (Table 1). Phylogenetic analyses of single-copy genes conserved among *Wolbachia* strains confirmed that the *w*Fem strain belonged to the supergroup B-type *Wolbachia* – which includes the feminizing strain *w*VulC found in *A. vulgare* (Fig. 1a). However, *w*VulC and *w*Fem were not closely related (ANI: 0.902) and exhibited highly divergent genome structures (Fig. 1b), highlighting the distinct evolutionary processes of feminizing *Wolbachia* in arthropods. In contrast, *w*Fem and *w*CI in *Eurema* shared more co-linear genomes (ANI: 0.954) and had comparable genome sizes and numbers of protein-coding genes (Fig. 1c and Table 1).

**Table 1.** Genome information of *w*Fem and two *Wolbachia* strains used for genome comparison in the dot plots shown in Fig. 1b, c

Strain	*w*Fem	*w*CI*	*w*VulC†
Sequence length (bp)	1,363,402	1,358,360	1,711,483
Number of contigs	1	1	1
G+C content (mol%)	33.9	34.1	34.9
No. of CDSs	1,307	1,249	1,666
No. of rRNAs	3	3	3
No. of tRNAs	34	33	35
Accession no.	AP038754	AP028951	CP156068.1
Host	*E. mandarina*	*E. mandarina*	*A. vulgare*
Phenotype	Feminization	Cytoplasmic incompatibility	Feminization

*Arai *et al.* [16].

†Grève *et al.* [59].

### Male-killing genes and male-killing-associated prophages in *w*Fem

**Fig. 2.**
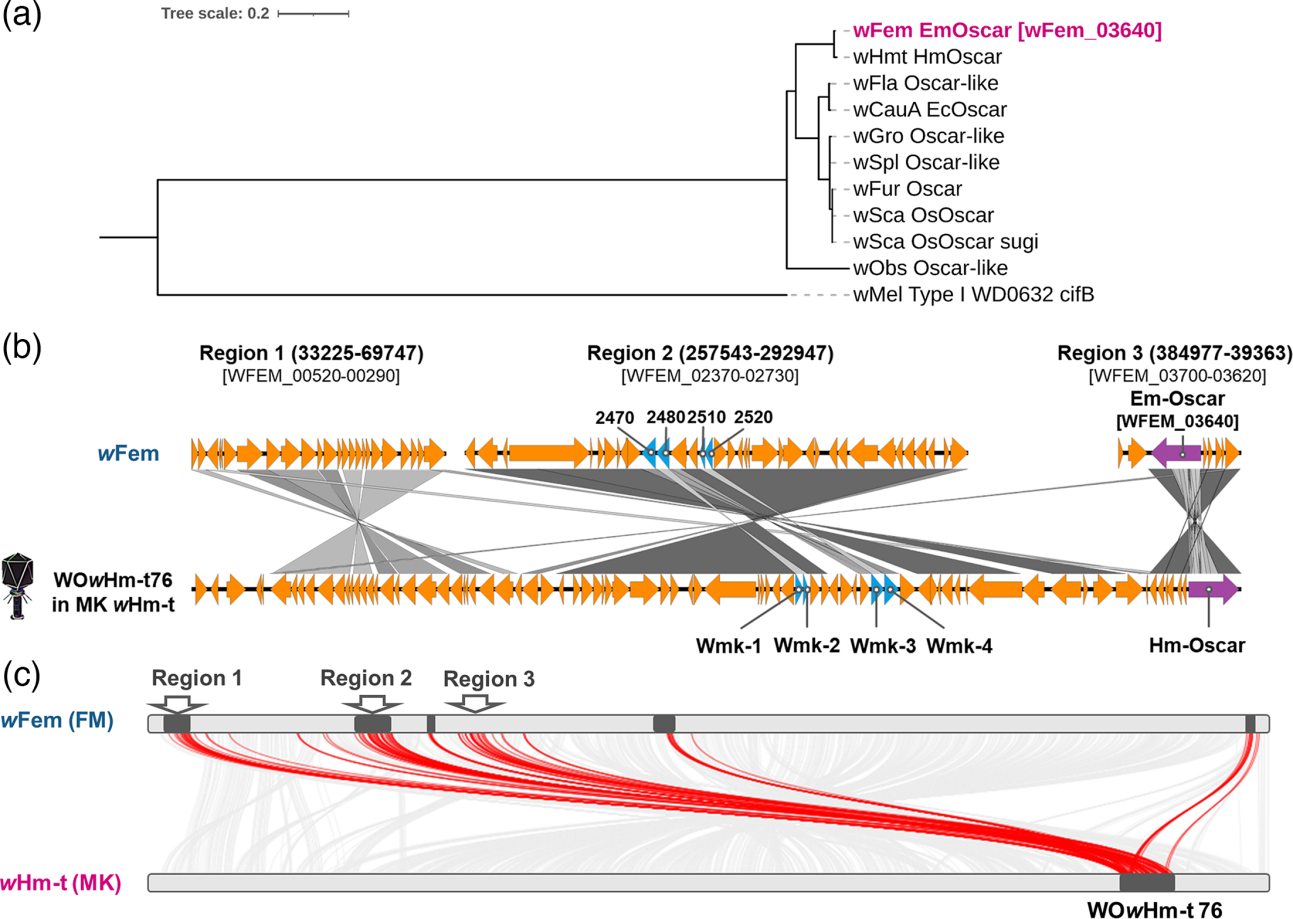
Male-killing genes and prophage-associated genomic regions in *w*Fem. (**a**) Phylogeny of the predicted amino acid sequences of Oscar homologues in *Wolbachia* strains. (**b**) Gene homologies between the prophage region WO*w*Hm-t76 from *w*Hm-t and those in the genome of *w*Fem. The *Em-oscar* gene (highly homologous to *Hm-oscar*) and the four *wmk* genes (homologous to *wmk*-1 to *wmk*-4) are marked in purple and blue, respectively. (**c**) Homologies between the male-killing-associated prophage region WO*w*Hm-t76 of *w*Hm-t and the genome of *w*Fem. Potential prophage regions were annotated using PHASTEST (shown in black). Homologous regions are connected by red lines. Regions 1–3, which show the highest homology percentages, are magnified in panel (**b**).

*w*Fem encodes an intact *oscar* homologue (*Em-oscar*) and seven intact *wmk* homologues, which are associated with male lethality in lepidopterans and dipterans, respectively. *w*Fem also encoded type V *cifB* homologues but lacked *cifA*, *pif* and *piff* genes, which are associated with *Wolbachia*-induced cytoplasmic incompatibility and parthenogenetic phenotypes (Fig. 2a, b and Table S3). In addition, *w*Fem and *w*CI share two *wmk* homologues (WCI_02550 and WFEM_02480; WCI_03640 and WFEM_03750). *w*CI-encoded CifB (WCI_03500) and *w*Fem-encoded Em-Oscar (WFEM_03640) were annotated as orthologous protein clusters (i.e. not assigned as strain-specific genes by OrthoFinder), but their protein sequences were highly divergent except for the conserved 3′ regions encoding peptidase domains (identity: 37.183, bit score: 166, e-value: 9.17e^−45^, blastp). Remarkably, *Em-oscar* showed high nucleotide sequence similarity with *Hm-oscar* from the male-killing *Wolbachia* strain *w*Hm-t (an endosymbiont of the moth *Homona magnanima*) (identity: 99.6%, bit score: 6,468, e-value: 0, blastn) (Table S2). Notably, *Hm-oscar* (*Hb-oscar*) is also conserved in the genome of the male-killing *Wolbachia* strain *w*Bol1Y from the nymphalid butterfly *Hypolimnas bolina* [44].

*Hm-oscar* is located in the male-killing-associated prophage region WO*w*Hm-t76 of *w*Hm-t [26]. Similarly, *w*Bol1Y encodes the *Hb-oscar* gene in its prophage region, which is highly similar to the WO*w*Hm-t76 region of *w*Hm-t [44]. Although *oscar* homologues generally exhibit high sequence diversity and vary considerably in length [16], male-killing *w*Hm-t, male-killing *w*Bol1Y and feminizing *w*Fem share a highly homologous *oscar* gene despite their phylogenetic distance (Figs 1a and 2a). This prompted us to investigate the prophage regions in the *w*Fem genome. Notably, *w*Fem encodes prophage regions similar to those of WO*w*Hm-t76 (Fig. 2b, c). Whilst the WO*w*Hm-t76-like regions in *w*Fem were fragmented into multiple segments, some of which likely represent prophage remnants, the genes adjacent to the *Em-oscar* (*w*Fem) and *Hm-oscar* (*w*Hm-t) were highly conserved (Fig. 2b). Given that *w*Fem and *w*Hm-t are distantly related (Fig. 1a), phage movement may have played a role in the evolution of both the feminizing and male-killing *Wolbachia* strains. Although the ecology of phage WO remains poorly understood, *Homona* moths and *Eurema* (and *Hypolimnas*) butterflies occur sympatrically across East and Southeast Asia, providing potential opportunities for horizontal transfer between their associated *Wolbachia* strains.

### Comparative genomics of *w*Fem and *w*CI in *Eurema* butterflies

In addition to *Em-oscar*, we identified 100 *w*Fem-specific genes that were absent in *w*CI (Tables S4 and S5). Most *w*Fem-specific genes encode hypothetical proteins, but some of the encoded eukaryotic motifs, such as ankyrin repeat domains, have been annotated as potential virulence factors linked to the host immune system [virulence factors BrkB (WFEM_09550) and TrbC/VIRB2 pilin (WFEM_09560)]. *w*Fem also encoded 25 strain-specific gene clusters involving transposases (*n*=13), hypothetical proteins (*n*=5) and genes encoding ankyrin repeat domains (*n*=2) (Table S5). Although the functions of these *w*Fem-specific genes remain unclear, they may be involved in the *w*Fem-induced phenotypes in *Eurema* butterflies.

In contrast to *w*CI, *w*Fem did not encode *cif* gene pairs in its genome. *w*Fem-singly infected individuals are rarely observed in nature, likely because of cytoplasmic incompatibility, which leads to embryonic lethality in the offspring of *w*Fem-infected females and *w*CI-infected males. As long as cytoplasmic incompatibility-inducing *w*CI is present in the population, *w*Fem, lacking the gene *cifA* responsible for rescuing cytoplasmic incompatibility, likely has no means of persisting other than through co-infection with *w*CI.

### Evolution of male killing and feminizing *Wolbachia* in lepidopteran insects

The male-killing genes *wmk* and *oscar* in the genome of feminizing *Wolbachia w*Fem postulate mechanistic links between *Wolbachia*-induced phenotypes. *Oscar* homologues appear to play a predominant role in the male-killing phenotype in Lepidoptera, as male killing is almost invariably observed in cases where *Oscar* is present [16,24, 26, 44, 46]. Although the male-killing function of *wmk* has been experimentally demonstrated only in *Drosophila melanogaster*, its functional relevance in lepidopteran hosts remains largely unknown [23,26, 47,50]. Nevertheless, some lepidopteran *Wolbachia* strains that lack *Oscar* but carry *wmk* also induce male killing [44], suggesting that a potential contribution of *wmk* to this phenotype cannot be entirely ruled out. Given that *wmk* is widely conserved among *Wolbachia* strains and often occurs in multiple copies regardless of the presence or absence of a male-killing phenotype, it may contribute more broadly to *Wolbachia* biology beyond male killing, including in the *w*Fem strain.

In some moth species, male-killing *Wolbachia* induces female-type sex determination in male hosts [14,15, 51]. *oscar* homologues of *w*Fur (*oscar*) and *w*Hm-t (*Hm-oscar*) induce male killing in their native hosts by suppressing the function of the upstream sex determination factor Masculiniser, which leads to the failure of the dosage compensation and the expression of female-specific splicing patterns of the downstream sex determinant *doublesex* in males [24,26, 46, 52]. Furthermore, recent studies have shown that *oscar*-bearing *Wolbachia* [*w*Sca (male killing in *Ostrinia*), *w*Hm-t (male killing in *Homona*), *w*CauA (male killing in *Ephestia*), *w*Bol1Y (male killing in *Hypolimnas*) and *w*Fem (feminization in *Eurema*)] generally ‘feminize’ sex determination cascades in cells derived from male *Ostrinia* moths [16]. Despite the high sequence diversity of known *Oscar* homologues [16], the *w*Fem-encoded *Em-oscar* shared 99.6% nucleotide sequence identity with the *Hm-oscar* gene, which is responsible for *w*Hm-t-induced male killing in *Homona* moths [46]. These findings suggest common mechanisms underlying *Wolbachia*-induced male killing and feminization in lepidopterans. In addition, *oscar* homologues were not identified in the genome of the feminizing *Wolbachia w*VulC found in *A. vulgare*, implying that *Wolbachia* have evolved multiple feminizing mechanisms in arthropods (insects and isopods), possibly reflecting their distinct sex determination systems.

*w*Fem, *w*Hm-t and *w*Bol1Y are phylogenetically distantly related but share highly homologous male-killing-associated genes (i.e. *oscar* and *wmk*) and prophage regions. Although the evolutionary origin of male-killing-associated prophage regions in phylogenetically distinct *Wolbachia* remains unclear, this study suggests that phage transfer may underpin the evolution of male killing and feminizing *Wolbachia* in Lepidoptera. Kageyama *et al.* [11] hypothesized an evolutionary transition from canonical male killing to feminization. In *Homona* and *Hypolimnas*, *oscar*-bearing *Wolbachia* induce male killing, and the removal of *Wolbachia* results in a normal sex ratio [44,53]. In contrast, removal of the *oscar*-bearing *Wolbachia* in *Ostrinia* and *Eurema* results in ‘female killing’ [6,54], suggesting that females require *Wolbachia* to survive due to the loss of the feminizing elements (W chromosome function in *Ostrinia* and W chromosome per se in *Eurema*). The redundancy in the feminizing functions of both the W chromosome and *Wolbachia* may have led to the loss of the W chromosome in some insects [11]. *oscar*-bearing *Wolbachia* are likely to have undergone an evolutionary transition from facultative male-killing parasites to obligate feminizers by driving the evolution of the sex determination system in insects.

However, *oscar*-based feminization and loss of the W chromosome may not be sufficient to produce a drastically female-biased sex ratio in butterflies in nature. Genetic males (ZZ) infected with *oscar*-bearing *Wolbachia* cannot survive as functional females [11,44, 51, 53], probably due to the failure of dosage compensation [24,46]. If the benefits of male killing in the host do not outweigh the costs of infection, *Wolbachia* would not spread in the host population, given that *Wolbachia* is typically transmitted vertically (maternal transmission) but rarely horizontally. To maximize the transmission efficiency, *Wolbachia* had to develop a novel method for producing functional females in the host. The combined actions of feminization (a common mechanism of male killing) and the selective production of Z0 individuals (potentially ZW in the past) – accomplished by inhibiting the maternal transmission of Z chromosomes – probably ensured the rapid and massive expansion of *w*Fem in *Eurema* butterflies, as observed in nature [5,55]. While the mechanisms (causative genes) underlying *w*Fem-induced blocking of the maternal inheritance of Z chromosomes remain largely unclear, it is likely that *w*Fem evolved this phenotype independently of the feminization acquisition process.

## Concluding remarks

In this study, we highlighted the common evolutionary history of male killing and feminizing *Wolbachia* by analysing the complete genome of *w*Fem. Prophages carrying genes associated with virulence and reproductive manipulations (such as *cif*, *wmk* and *oscar*) probably underpin the evolution of male killing and feminizing *Wolbachia* in insects. Closely related *Wolbachia* strains sometimes co-infect a host [6,56,58], which has hindered our understanding of their genetic bases. Single-cell genomics will shed light on the mechanisms underlying host manipulation by uncultivable endosymbionts such as *Wolbachia*. Further comparative genomics, trans-infection assays and functional validations will provide valuable insights into the mechanisms underlying *Wolbachia*-induced phenotypes and their evolutionary histories.

## Supplementary material

10.1099/mgen.0.001578 Uncited Supplementary Material 1.

## Abbreviations

ANI: average nucleotide identity
